# The Budget

**Published:** 1920-05-01

**Authors:** 


### The Hospital

The Workers' Newspaper of Administrative Medicine and Institutional Life, Administration, National Insurance and Health.

No. 1769, Vol. LXVIII. SATURDAY, MAY 1, 1920. PRICE TWOPENCE

### THE BUDGET.

From the especially institutional, as opposed to the individual, standpoint, this year's Budget is comparatively colourless, staggering though the figures of our national indebtedness and our national taxation must appear to all of us in our capacity as citizens. No sympathy has been extended to the suggestion that donations and subscriptions to approved charitable institutions of the voluntary hospital type shall be allowed to be deducted from the donor's statutory income for taxation purposes —a suggestion which has on several occasions been commended in these columns as one likely to ease a small part of the financial stress now threatening a large proportion of the voluntary hospitals of the country. The proposed five per cent. tax upon the profits of trading corporations, which may or may not survive the criticisms to which it is being subjected, has no direct effect upon hospitals, except in so far as it may conceivably tend to cause yet further rises in the price of the commodities which every hospital has to buy. The restoration of the excess profits tax to a higher level is of the same order: except as it may conduce to profiteering it has no incidence upon the funds of charitable institutions.

The comprehensive and long overdue reform of the income tax affects many individuals, some by way of relief, others by way of the reverse. What effect these changes may have upon the springs of private charity or upon the testamentary dispositions of benefactors by will is impossible to foretell: probably, very little indeed. In another column (p. 115) will be found a brief summary of the newly amended income tax proposals, which have this great advantage over the old ones of being much simpler and less intricate. The summary in question deals with the matter mainly from the point of view of the professional man; but the tax as now to be arranged catches all alike, and the provisions apply to every kind of income-possessor, professional or otherwise, and as such are of general interest. It has been objected against these proposals that they tend still further to divorce taxation from representation; that is, that they accentuate the already existing state of affairs whereby the electoral power resides in the hands of voters only about one-tenth of whom pay any direct taxation. That this is a serious blot on the existing system of taxation can scarcely be denied; obviously it makes directly for national extravagance, for demagogic appeals to the demon of cupidity, and against national steadfastness and thriftiness. Otherwise, the new taxation is on the whole well devised and has found few opponents.

The new motor-car taxes are more controversial. The daily press has already shown that they penalise the careful owner who economises over petrol and tyres as far as he may, while favouring at his expense the driver who uses far more petrol than he need do. This is surely a very grave defect indeed, especially as motor spirit is an imported article, the undue use of which tends to increase the unfavourable exchange from which we are at present suffering.

The chief grievance of the medical profession, however, is that the rebate of half the licence and petrol duties hitherto granted has been taken away, or rather is proposed to be taken away. Questioned in Parliament, the Chancellor of the Exchequer has replied by promising to refer the matter elsewhere, which hardly looks promising for the apprehensions of the doctors. The great argument in favour of remitting this heavy taxation on medical automobiles is that a doctor with a car can do at least twice the work in a day that a doctor with a horsed carriage can. Why, therefore, should the State penalise the man who is twice as productive as the other, and damages the roads less? It is directly to the advantage of the community that the work required of the medical profession should be carried out by the smallest number of men (and women) who can do it efficiently; for thus others are set free for labours more directly productive (not more productive indirectly, of course), to the advantage of the State. The same argument applies, for the same reasons, which may be stated briefly as the saving of man-power, to motor vehicles used for trade purposes, *e.g.*, by commercial travellers. It remains to be seen whether the profession will be able to press this point of view successfully on the Chancellor and the Revenue authorities.

One more point in connection with the motor taxes is the question of the motor ambulances used by hospitals or by local authorities. It is to be hoped someone is watching this from the point of view of the voluntary hospitals, and that the Legislature will in its wisdom see fit to exempt such cars from the heavy taxation now proposed to be levied on other car owners.

